# Evaluation of asthma–chronic obstructive pulmonary disease overlap using a mouse model of pulmonary disease

**DOI:** 10.1186/s12950-022-00322-x

**Published:** 2022-12-06

**Authors:** Yong Suk Jo, Chin Kook Rhee, Hyoung Kyu Yoon, Chan Kwon Park, Jeong Uk Lim, An Tai Joon, Jung Hur

**Affiliations:** 1 Division of Pulmonary and Critical Care Medicine, Department of Internal Medicine, Seoul St. Mary’s Hospital, College of Medicine, The Catholic University of Korea, Seoul, South Korea; 2 Division of Pulmonary and Critical Care Medicine, Department of Internal Medicine, Yeouido St Mary’s Hospital, College of Medicine, The Catholic University of Korea, Seoul, South Korea

**Keywords:** Asthma, Chronic obstructive pulmonary disease, Experimental model, Cytokine, Lung function

## Abstract

**Background:**

Features of asthma and chronic obstructive pulmonary disease (COPD) can coexist in the same patient, in a condition termed asthma– chronic obstructive pulmonary disease overlap (ACO). ACO is heterogeneous condition exhibiting various combinations of asthma and COPD features. No clinically acceptable experimental model of ACO has been established. We aimed to establish an animal model of ACO.

**Methods:**

We generated two phenotypes of ACO by administering ovalbumin and porcine pancreatic elastase in combination, and papain. The proinflammatory cytokines and cell types in bronchoalveolar lavage fluid (BALF) were investigated, and lung function parameters were measured using the FlexiVent system.

**Results:**

Greater airway inflammation was observed in the asthma and both ACO models, and emphysema was found in the COPD and both ACO models. The proportion of eosinophils in BALF was elevated in the asthma and ACO-a model. Type 2 inflammatory cytokine levels were highest in the ACO-a model, and the neutrophil gelatinase–associated lipocalin level was elevated in the asthma and ACO-a model. Of lung function parameters, compliance was greater in the COPD and ACO-b model, in which elastance was lower than in the asthma model. Airway resistance increased with the methacholine concentration in the asthma and both ACO models, but not in the control or COPD model.

**Conclusion:**

We established two murine models of ACO that exhibit features of asthma and COPD. We validated the clinical relevance of the ACO models based on changes in cytokine profiles and lung function. These models will be useful in further studies of the pathogenesis of, and therapeutic targets for ACO.

**Supplementary Information:**

The online version contains supplementary material available at 10.1186/s12950-022-00322-x.

## Background

Asthma and chronic obstructive pulmonary disease (COPD) were formerly regarded as distinct diseases with different pathophysiologies [1, 2]. Since Gibson and Simpton [3] first described asthma– chronic obstructive pulmonary disease overlap (ACO), which has the clinical features of asthma and COPD simultaneously, little information on ACO has been obtained despite of growing interests and much research. Moreover, the differentiation of ACO from asthma or COPD in patients is challenging because of the lack of consensus on diagnostic criteria; indeed, subjects with ACO have been mutually excluded from clinical studies of asthma and COPD [4]. Therefore, the prevalence of ACO has been reported with wide range; it was reported 11.1-61.0% among asthmatics and 4.2-66.0% in patients with COPD [5].

Relative to patients with asthma or COPD alone, individuals with ACO have more severe respiratory symptoms, poorer self-perceived quality of life, and reduced lung function; they experience exacerbations more frequently and have higher mortality rates [6–11]. Given the lack of consensus on the diagnostic definition of ACO, the reported prevalence, clinical features, and outcomes of this condition are diverse and depend on cohort characteristics and the diagnostic criteria used. Similar prevalence and clinical features of ACO were reported in two large COPD cohorts when identical diagnostic criteria were applied [12]. In addition, no effective treatment for ACO is available. ACO is a complex, heterogeneous condition, and animal studies are required to elucidate its pathophysiological mechanism.

Animal studies of ACO are scarce, and the establishment of ACO models with pathophysiological features of asthma and COPD is challenging. Porcine pancreatic elastase (PPE) has been used to induce emphysema as a murine model of COPD [13], and ovalbumin (OVA) in the presence of an adjuvant, typically aluminum hydroxide (alum), has been used to induce experimental allergic asthma [14]. Intra-tracheal aerosol administration of papain induced not only macrophage/neutrophilic but also type 2 inflammation–associated cytokine expression and emphysema [15]. Here, we established a murine model of ACO using PPE with OVA/alum or papain and evaluated cytokine profiles, airway inflammation, and lung function.

## Methods

### Mice and Model establishment

Female C57BL/6N mice (Orient, Gyeongi-do, Korea) aged 6 weeks were used in this study. The mice were divided randomly into control, asthma, COPD, ACO-a, and ACO-b groups.

**Fig. 1:**
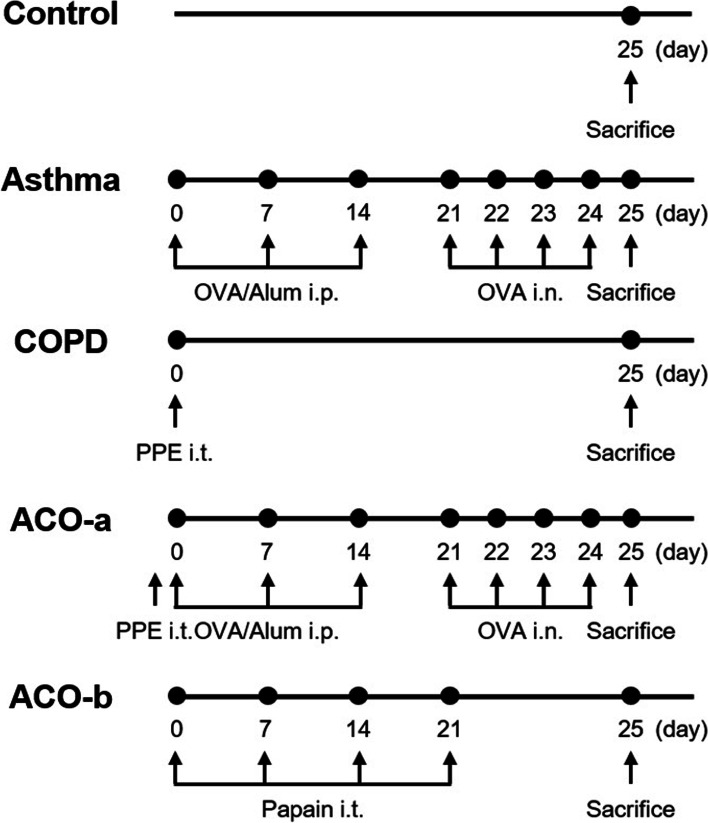
Pulmonary disease models. ACO, asthma–chronic obstructive pulmonary disease overlap; alum, aluminum hydroxide; i.p., intraperitoneal; i.t., intratracheal; OVA, ovalbumin; PPE, porcine pancreatic elastase

Mice in the asthma group were immunized with 50 μg OVA (chicken egg albumin, grade V; Sigma-Aldrich, St. Louis, MO, USA) in 1 mg aluminum hydroxide (Sigma-Aldrich) in 200 μL phosphate-buffered saline (PBS). Immunization was performed by intraperitoneal injection on days 0, 7, and 14, and intranasal OVA challenges (100 μg/50 μL PBS) were administered on days 21, 22, 23, and 24 under anesthesia with isoflurane (Vedco, St. Joseph, MO, USA). Mice in the COPD group received intratracheally administer PPE (80 U/kg; Elastin Products Company, Owensville, MI, USA) in 100 μL PBS on day 0. Mice in the ACO-a group received the treatments administered to the asthma (OVA) and COPD (PPE) groups. Mice in the ACO-b group were treated intratracheally with 50 μg papain (Sigma-Aldrich) in 100 μL PBS on days 0, 7, 14, and 21. PPE or papain aerosol was created using a Micro Sprayer Aerosolizer (Penn Century Inc., Wyndmoor, PA, USA). The animals were euthanized on day 25 (Fig. 1).

### Respiratory mechanics

Respiratory mechanics were analyzed using a FlexiVent system (SCIREQ, Montreal, QC, Canada). The mice were weighed and anesthetized by intraperitoneal injection of a zoletil-rompun mixture (3:1). The trachea was exposed and cannulated, and the animal was connected to a computer-controlled small-animal ventilator and ventilated with the following settings to achieve a mean lung volume close to that occurring during spontaneous breathing: tidal volume of 10 mL/kg, frequency of 150 breaths/min, and positive end-expiratory pressure of 2 cmH_2_O. Next, a “snapshot perturbation” maneuver was performed to measure the resistance (R), elastance (E), and compliance (C) of the respiratory system. The forced oscillation perturbation “primewave-8” was consequently applied, yielding airway resistance (Rn), tissue damping, and tissue elasticity (H).

After performing all perturbations at a baseline level, a previously described protocol for the measurement of airway hyper-responsiveness (AHR) was initiated [16].

### Blood and bronchoalveolar lavage fluid collection

Immediately after AHR measurement, blood was collected from the jugular vein, clotted, and centrifuged at 4,000 rpm for 10 min. The serum was stored at –80°C.

Bronchoalveolar lavage fluid (BALF) was collected immediately after AHR measurement. The exposed trachea was cannulated with silicone tubing attached to a 22-guage needle on a 1-mL tuberculin syringe. BALF was collected after the instillation of 0.8 mL sterile PBS and centrifuged at 3,000 rpm for 5 min at 4°C. The supernatants were collected and stored at –80°C. The total cell counts in BALF were obtained using the LUNA^TM^ automated cell counter (Logos Biosystems, Inc., Annandale, VA, USA). The BALF was centrifuged onto microscope slides at 2,000 rpm for 7 min in a Cytospin centrifuge (Thermo Fisher Scientific, Waltham, MA, USA) and stained with a Shandon Kwik-Diff™ kit (Thermo Fisher Scientific). Macrophages, eosinophils, lymphocytes, and neutrophils in BALF were enumerated by counting 500 leukocytes in randomly selected fields under light microscopy.

### Enzyme-linked immunosorbent assay

The concentrations of immunoglobulin E (IgE; Invitrogen, Carlsbad, CA, USA) and neutrophil gelatinase–associated lipocalin (NGAL) in serum and of interleukin (IL)-4, IL-13, IL-17, and tumor necrosis factor-α (TNF-α) in BALF were measured using ELISA kits (R&D Systems, Minneapolis, MN, USA) according to the manufacturer’s instructions.

### Lung histopathology

Lung samples were fixed in 4% paraformaldehyde and embedded in paraffin wax. Sections were cut at 4 μm thickness using a microtome, and deparaffinized tissue sections were stained with hematoxylin and eosin to detect cellular infiltration. For the scoring of airway inflammation, the slides were numbered randomly and evaluated independently by two blinded investigators. The quantity of peribronchial or perivascular inflammation was assessed as described previously [17]. The mean linear intercept was calculated by measuring the alveolar diameter in 10 random fields per slide using a slide scanner (Pannoramic MIDI, 3DHISTECH Ltd. Budapest, Hungary, as described elsewhere [18].

### Statistical analysis

Data are presented as means ± standard errors of the mean. Data were compared between groups by one-way analysis of variance (ANOVA) with the *post hoc* Tukey test or two-way ANOVA with the Bonferroni test using the GraphPad Prism statistical software (GraphPad Software, Inc., San Diego, CA, USA). *P* values < 0.05 were considered to indicate significance.

## Results

### Body weight

The body weights of the mice are presented in additional Figure 1. The body weight tended to be greater, but not significantly so, in the asthma, COPD, and ACO groups compared with the control.

### BALF leukocyte composition

**Fig. 2:**
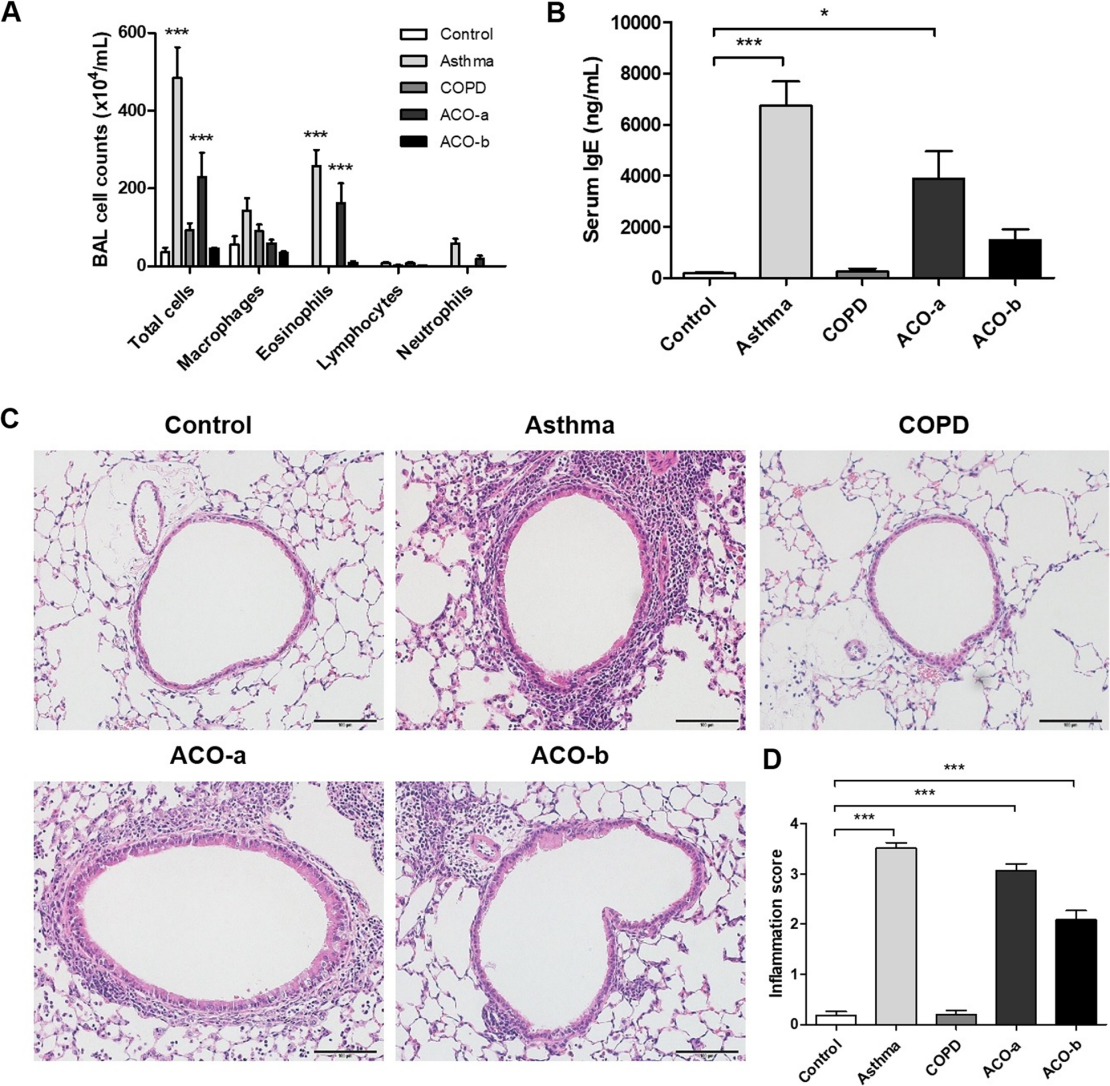
Airway inflammation. **A** Differential cell counts in bronchoalveolar lavage fluid (BALF), **B** serum total IgE concentrations, **C** lung tissue stained with hematoxylin and eosin (20×), **D** inflammation scores. ***p < 0.001 vs. control

Compared with the control, the leukocyte and eosinophil numbers in BALF were significantly greater in the asthma and ACO-a models (Fig. 2A). The serum total IgE level was markedly increased in mice in the asthma and ACO-a groups compared with the control (Fig. 2B). Furthermore, inflammatory cell aggregates (Fig. 2C) and inflammatory scores (Fig. 2D) were increased in the asthma and both ACO models.

### Inflammatory cytokines

**Fig. 3:**
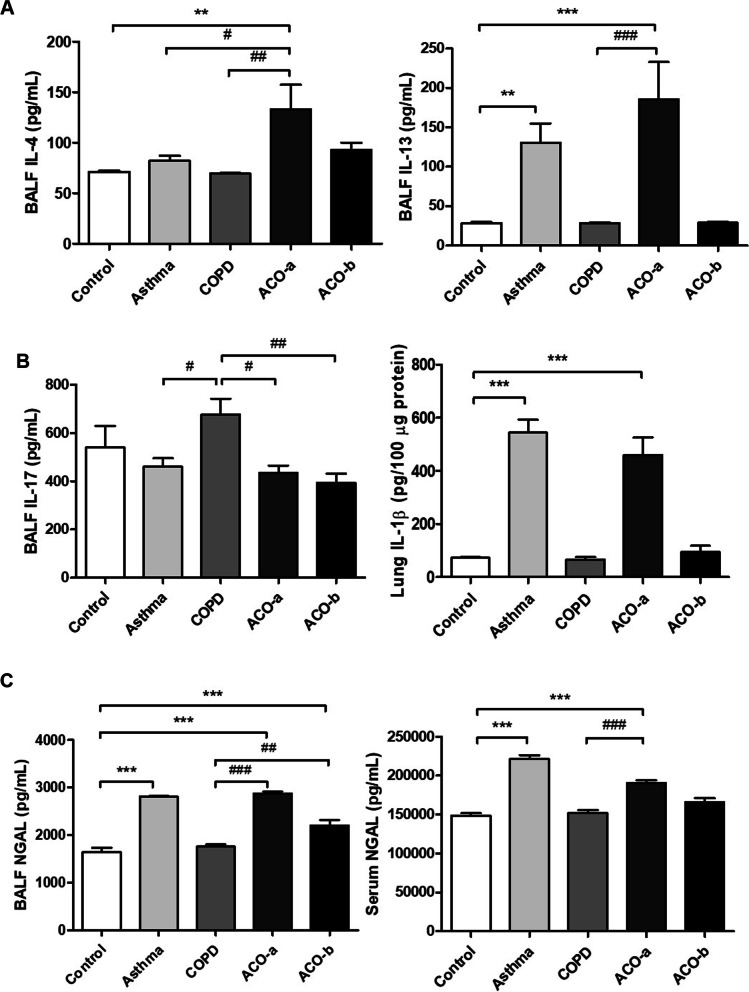
Inflammatory cytokines. **A** Type-2 inflammatory cytokines in BALF, **B** macrophage/neutrophil-associated inflammatory cytokines in BALF and serum, **C** NGAL in BALF and serum. ^*^vs. control, ^#^vs. asthma; ^*^p < 0.05, ^**^p < 0.01, ^***^p < 0.001

The IL-4 level was higher in the ACO-a model than in mice with asthma and COPD, and the IL-13 level was higher in the asthma and ACO-a models than in the COPD and ACO-b models (Fig. 3A). By contrast, the IL-17 level was higher in the COPD model than in the asthma and both ACO models (Fig. 3B). However, the IL-1β level in lung tissue homogenates and the TNF-α level in BALF were significantly higher in the asthma and ACO-a models than in the control. The TNF-α level was higher in the ACO-a model than in the asthma model (Additional Fig 2).

The NGAL level in BALF was higher in the asthma and both ACO models than in the control, and higher in both ACO models than in the COPD model (Fig. 3C). The serum NGAL level was higher in the asthma and ACO-a models than in the control and COPD models.

The IL-6 level was significantly increased in the asthma and ACO-a models compared with the control and COPD models. In the ACO-b model, the IL-6 level was non-significantly elevated relative to those in the control and COPD models (Additional Figure 2).

### Lung function

**Fig. 4:**
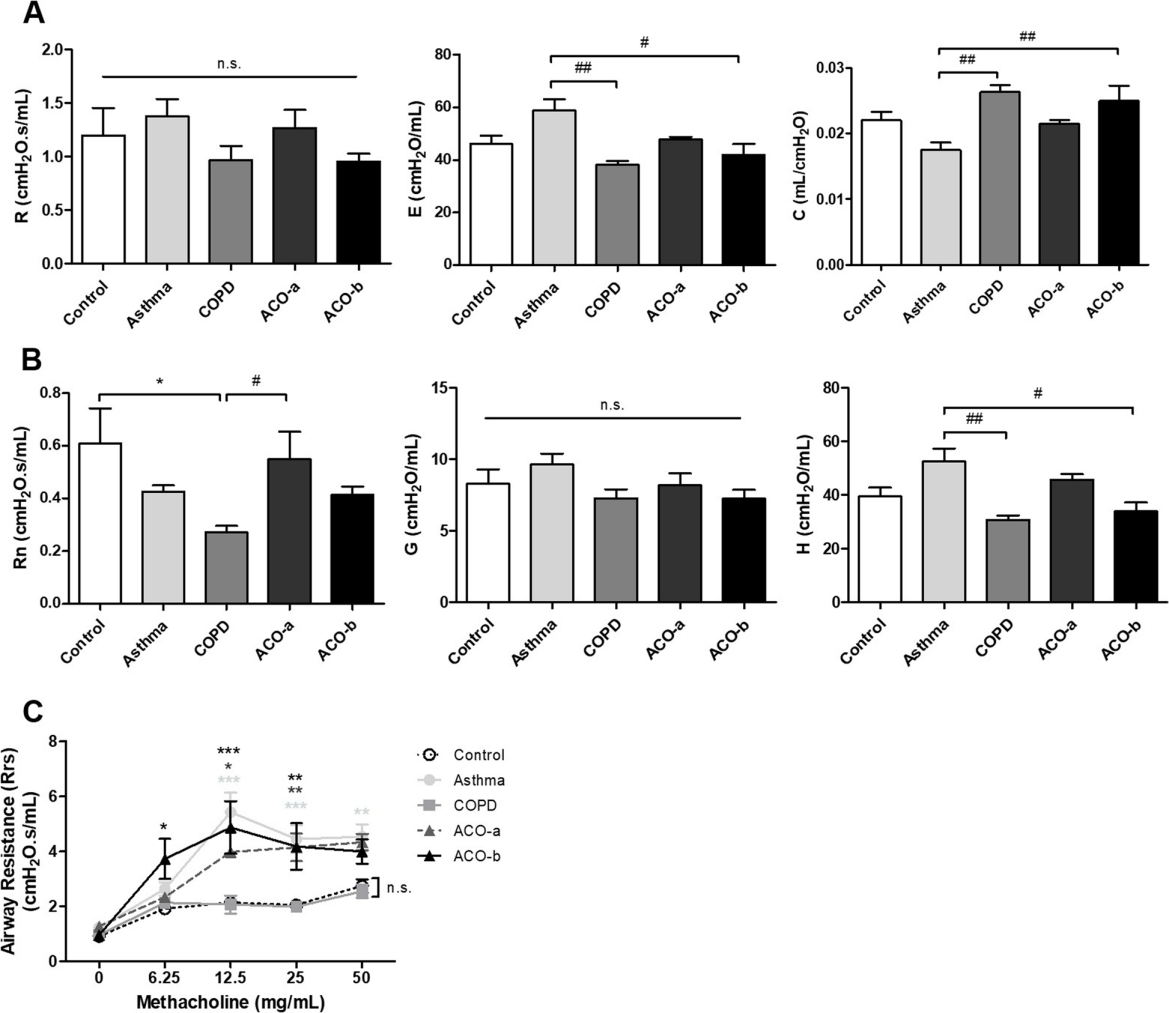
Respiratory mechanics. (A) Snapshot perturbation; resistance (R), elastance (E), and compliance (C). As the lung is seen as a single compartment, the parameters (R, E, and C) are indicative of the whole thorax (chest wall and lung). (B) Primewave-7 perturbation; airway resistance (Rn), tissue damping or resistance (G), and tissue elasticity (H). As the lung is seen as multiple compartments, Rn, G, and H can differentiate airways. (C) Airway responsiveness to increasing doses of methacholine measured by airway resistance. ^*^vs. control, ^#^vs. asthma; ^*^p < 0.05, ^**^p < 0.01, ^***^p < 0.001; n.s., not significant

R did not differ between the four models and the control. E was significantly reduced and C was significantly increased in the COPD and ACO-b models compared with the asthma model (Fig. 4A).

A primewave-8 perturbation model showed significantly decreased Rn in the COPD model compared with the control and decreased H in the COPD model compared with the asthma model. The Rn was significantly increased in the ACO-a model compared with the COPD model, and the H was significantly decreased in the ACO-b model compared with the asthma model (Fig. 4B).

Regarding AHR, methacholine significantly increased airway resistance in the asthma and both ACO models, but did not affect the control or COPD model (Fig. 4C).

### Extent of emphysema

**Fig. 5:**
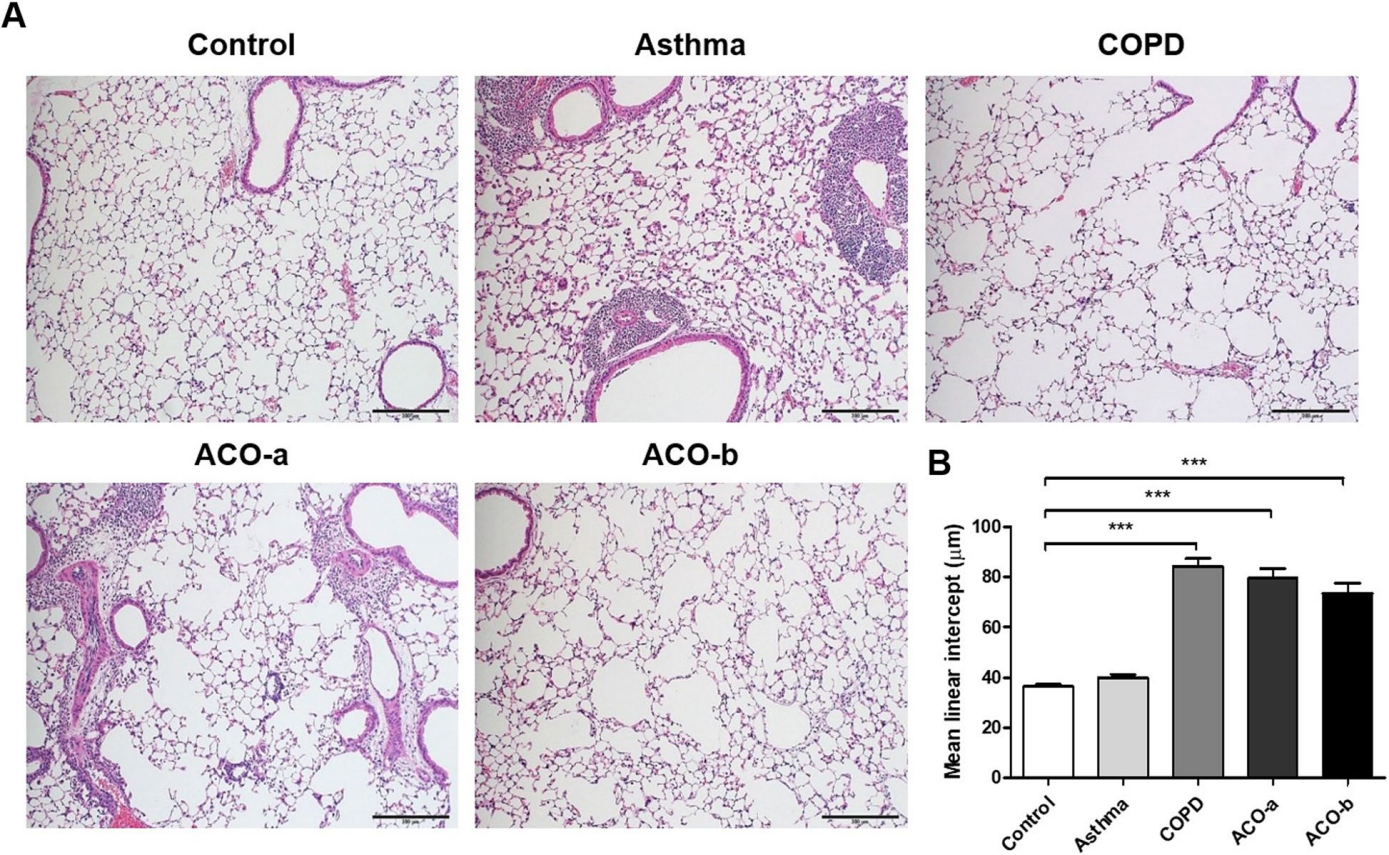
Lung tissue (**A**) from the airway models stained with hematoxylin and eosin (10×) to evaluate air space widening (emphysema). (**B**) Mean linear intercepts, calculated by measuring alveolar space diameters in random fields per slide using a slide scanner (Panoramic MIDI)

Histological analysis revealed air space widening in lung tissue in the COPD and both ACO models, but not in the asthma model (Fig. 5A). Moreover, the mean linear intercept was significantly increased in the COPD and both ACO models (Fig. 5B).

## Discussion

We established two distinguishing mouse models of ACO that exhibited features of asthma and COPD using PPE with OVA and papain, and subjected them to comprehensive analysis. The mouse models were acceptable and clinically suggestive of ACO. They will be useful for the investigation of the pathogenesis of ACO and development of diagnostic markers and therapeutic targets.

Prior experimental models of ACO involved exposure to allergens and cigarette smoke (CS) [19–21], which does not reflect the pathogenesis of ACO and there are remain unmet needs for experimental models of ACO because of some barriers. To set up appropriate animal models that best reflect the pathogenesis of ACO, combination of the most relevant features from both experimental model of asthma and COPD will enable us to make the acceptable animal model for ACO. Exposure to OVA or house dust mites is used to induce airway inflammation and remodeling in asthma models [22]. The classical mouse model of COPD makes use of lipopolysaccharide, PPE, elastase, and cigarette smoke extract (CSE) [23]. The intraperitoneal injection or inhalation of CSE can induce airway inflammation [24, 25]. However, exposure to CSE and allergens does not yield consistent airway inflammation and airway resistance [21, 26–28]. In one study, male surfactant protein-D gene deletion in C57BL/6J mice aged 8–10 weeks exposed to OVA and CS was used; type-2 inflammation did not differ between the OVA and OVA + CS groups, and CS-exposed mice failed to show emphysematous changes [19]. Thus, further studies are needed to develop a CSE exposure protocol and clarifying the role of CS exposure for development of ACO model.

Our pulmonary disease mouse model required only 3 weeks to establish after the administration of OVA with PPE and papain, and thus reflects early-onset airway inflammation. Emphysema developed in the COPD and ACO models, but macrophage/neutrophil-associated inflammatory cytokines were not consistently elevated in these models. This factor constitutes a limitation of our models, related to the consideration that emphysema in the ACO and COPD models was not developed over a long period or associated with smoking exposure; furthermore, emphysema alone is not representative of COPD. To establish model of COPD is complicated and difficult because COPD is composed of anatomical and functional components and it develops slowly and progressively over many years. There are few animal models of COPD related to emphysema, cigarette smoke exposure and other noxious particles exposure and even starvation model [13]. However, unfortunately none of models succeeds approximate the pathophysiological events occurred in human lung. Elastase induced emphysema model could not reflect the mechanisms actually occurring in human airways by smoke. However, smoking history is one of the feature implying COPD component, not mandatory. We focused on easier and simpler model and our emphysema COPD and ACO model does not require several months and special experimental equipment. Furthermore, our ACO model was set up in two separate ways and this might reflect the heterogeneity of ACO itself. In two nationwide COPD cohorts, the proportions of current smokers in the ACO groups were smaller than or similar to those in the COPD groups [12]. Although CS exposure is a risk factor for COPD, approximately 25–40% of COPD cases are not associated with smoking [29]. Our ACO models encompassed emphysema, but not airway inflammation consistent with COPD. The reflection of all COPD endotypes in an ACO model is difficult. However, our models are simpler to establish and more reproducible than are those based on CSE exposure.

Type 2 inflammation mediated by IL-4 or IL-13 and eosinophilic inflammation in blood or sputum are markers of an asthmatic endotype [30] and can be suppressed by targeted therapy [31]. We observed the elevation of eosinophils in BALF and type 2 cytokine expression in the ACO-a model; indeed, the levels were comparable to or higher than those in the asthma model. Some studies reported NGAL is a promising ACO-specific marker [32, 33]. The NGAL level in BALF was higher in both ACO models than in the COPD model, and the level of NGAL in serum was higher in the ACO-a model than in the COPD model. The BALF and serum NGAL levels were similar in the asthma and ACO-a models, suggesting that this biomarker can distinguish ACO from COPD. The blood eosinophil count is predictive of the response to inhaled corticosteroids (ICSs) in COPD [34–36]. Since the 2019 GOLD guidelines recommend that ICSs be used in combination with a long-acting bronchodilator for patients with COPD whose blood eosinophil counts ≥ 300/μL [2]. However, caution must be used with ICS inhaler treatment of COPD because of the variability in blood eosinophil counts and relevance to exacerbations [37]. Moreover, some patients with ACO do not benefit from ICS treatment [38], possibly because of the heterogeneity of ACO itself, just like asthma and COPD.

R was significantly decreased in the COPD model compared with the asthma and ACO models. E was significantly lesser and C was significantly greater in the COPD and ACO-b models than in the asthma model. E and C trends in the ACO-a model were similar to those in the COPD and ACO-b models. R increased with the methacholine concentration in the asthma and both ACO models. Thus, the physiological responses in the two ACO models reflect features of asthma (through AHR) and COPD (through simultaneously increased C and decreased E).

In clinical studies, obtaining consistent information for ACO patients was difficult because there are lack of consensus on unified diagnostic criteria and they are mutually excluded in both asthma and COPD trials. Also, ACO is considered as a heterogeneous condition and clinical presentation might be appeared differently depending on predominance of characteristics of either asthma or COPD [39]. We established two murine ACO models with the pathological features of asthma and COPD and confirmed their physiological characteristics. ACO-a model seems to have more asthmatic features, and ACO-b model seems to have more COPD features. Our ACO models reflected the heterogeneity of the disease and these can be used for better understanding of pathophysiology and facilitating further research on airway diseases. However, this study has several limitations. First, because the models were established 3 weeks after the administration of PPE with OVA and papain, they reflect early-onset inflammation. Follow-up studies on the long-term stability and reproducibility of the models are needed. Second, emphysema was induced rapidly and independent of smoke inhalation, and emphysema alone is not representative of COPD. Third, although AHR occurred in both ACO models, the magnitude of macrophage/neutrophilic and/or eosinophilic airway inflammation was not analyzed, especially in ACO-b models. Thus, efforts to develop a model that reflects neutrophilic inflammation and the clinical features of COPD beyond emphysema are warranted.

## Conclusion

ACO has a greater disease burden than does asthma or COPD alone. However, few animal models of ACO have been established because of the heterogeneity and complexity of the condition. We developed two ACO models with features of asthma and COPD. Our models are simple and easy to set up, and have high degrees of reproducibility. Thus, they will enable the investigation of the pathological mechanisms and responses to stimuli of ACO.

## Supplementary Information

**Additional file 1.**

## Data Availability

The datasets used and/or analyzed during the current study are available from the corresponding author on reasonable request.

## Abbreviations

ACO: Asthma– chronic obstructive pulmonary disease overlap
AHR: Airway hyper-responsiveness
BALF: Bronchoalveolar lavage fluid
COPD: Chronic obstructive pulmonary disease
NGAL: Neutrophil gelatinase–associated lipocalin
OVA: Ovalbumin
PPE: Porcine pancreatic elastase
