# Correction: Evaluation of asthma–chronic obstructive pulmonary disease overlap using a mouse model of pulmonary disease

**DOI:** 10.1186/s12950-023-00326-1

**Published:** 2023-01-20

**Authors:** Yong Suk Jo, Chin Kook Rhee, Hyoung Kyu Yoon, Chan Kwon Park, Jeong Uk Lim, Tai Joon An, Jung Hur

**Affiliations:** 1 Division of Pulmonary and Critical Care Medicine, Department of Internal Medicine, Seoul St. Mary’s Hospital, College of Medicine, The Catholic University of Korea, Seoul, South Korea; 2 Division of Pulmonary and Critical Care Medicine, Department of Internal Medicine, Yeouido St Mary’s Hospital, College of Medicine, The Catholic University of Korea, Seoul, South Korea

**Correction: J Inflamm 19, 25 (2022)**

**https://doi.org/10.1186/s12950-022-00322-x**

Following the publication of the original article [1], the authors identified an error in the author’s name of Tai Joon An. The given name and family name were erroneously transposed.

The incorrect author’s name is: An Tai Joon

The correct author’s name is: Tai Joon An

The author group has been updated above and the original article [1] has been corrected.
