# Supplementary material for: Evaluation of asthma–chronic obstructive pulmonary disease overlap using a mouse model of pulmonary disease
Source: J Inflamm (Lond). 2022 Dec 6;19:25. doi: 10.1186/s12950-022-00322-x (PMC9728005; doi:10.1186/s12950-022-00322-x)
Supplement: Supplementary file 1 — Additional file 1. [file 12950_2022_322_MOESM1_ESM.docx]

**Additional Figure legends**

**Additional Figure 1. Changes in body weight among airway models**

**
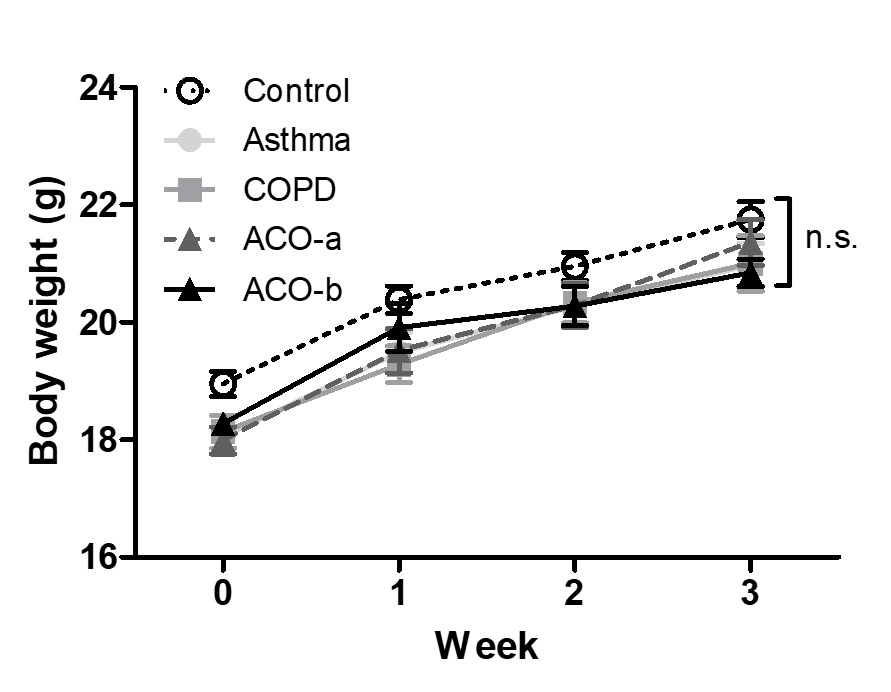
**

**Additional Figure 2. Inflammatory cytokines.** (A) Level of IL-6 in lung homogenates, (B) Tumor necrosis factor-α (TNF-α) in BALF.


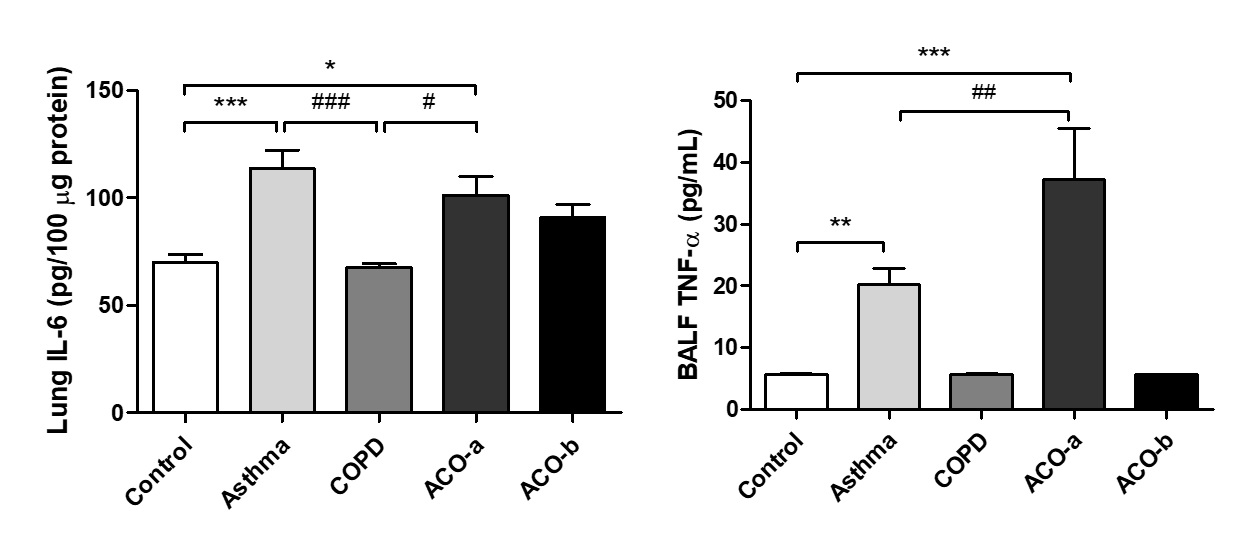


BALF, bronchoalveolar lavage fluid; IL, interleukin
